# The impact of industrial digitalization on the urban-rural income gap

**DOI:** 10.1371/journal.pone.0335065

**Published:** 2025-10-29

**Authors:** Xuan Lin, Yuantao Jiang

**Affiliations:** School of Economics and Management, Shanghai Maritime University, Shanghai, China; Peking University, CHINA

## Abstract

Along with the rapid development of the global digital economy, China is experiencing profound transformations in industrial digitization. These transformations may significantly affect the urban-rural income gap. Using panel data from 30 Chinese provinces from 2012 to 2022, this paper empirically examined the impact of industrial digitalization on the urban-rural income gap based on a fixed-effects model. The findings reveal that the development of industrial digitalization in China widens the urban-rural income gap. Mechanism analysis indicates that industrial digitalization increases software business revenue and employment in the information services sector, thereby expanding the urban-rural income gap; additionally, industrial digitalization widens the income gap between urban migrants and rural migrant populations, further increasing the overall urban-rural income disparity. Heterogeneity analysis demonstrates that in the eastern region, industrial digitalization significantly enlarges the urban-rural income gap, whereas its effects are not significant in the central and western regions. The conclusions of this study provide empirical support and policy insights for China in advancing industrial digitalization and promoting common prosperity.

## 1 Introduction

With the rapid development of digital technology, the digital economy has become a pivotal engine of global economic transformation. The digital economy grow primarily through two channels: digital industrialization and industrial digitalization (ID) [1]. As a core component of the digital economy, ID entails the deep integration of digital technologies with traditional industries [2]. It plays a vital role in improving production efficiency, promoting industrial upgrading, and driving socio-economic development [3]. ID not only influences overall economic growth but may also have a profound impact on narrowing or widening the urban-rural income gap.

The urban-rural income gap is a widespread challenge faced by both developed and developing countries around the world [4]. According to data from the Organisation for Economic Co-operation and Development (OECD, 2022), rural residents in many advanced economies continue to earn significantly less than urban counterparts while facing constrained access to digital services, educational resources, and quality employment opportunities [5]. In emerging countries such as India and Brazil, these disparities are frequently amplified by infrastructural deficiencies and highly informal labor market [6]. To address this global challenge, multiple nations have adopted digitalization strategies targeting urban-rural convergence. For example, India’s “Digital India” initiative seeks to improve digital infrastructure in rural areas, while mobile platforms like M-Pesa in East Africa have made notable progress in promoting financial inclusion [7]. However, the effectiveness of these initiatives varies considerably across countries, influenced by factors such as institutional environments, infrastructure quality, and labor market structures. These divergent findings necessitate further investigation into the impact of ID on income distribution across heterogeneous national contexts—notably in major emerging countries like China, where the urban-rural income gap remains pronounced.

Nonetheless, these global efforts often yield mixed results and are highly contingent on specific contextual conditions. The urban-rural income gap has long been a prominent issue in China’s socio-economic development. Although China has made progress in promoting urban-rural integrated development and reducing regional disparities, imbalanced regional development continues to sustain income divides [8]. With the advancement of ID, how to leverage this emerging force to narrow the urban-rural income gap has become an urgent and critical issue. By enhancing traditional industry efficiency and promoting coordinated regional economic development, ID may offer novel ideas and approaches for solving the urban-rural income disparity [9]. Consequently, exploring the impact and mechanisms of ID on the urban-rural income gap not only holds significant theoretical importance but also provides practical evidence for policy formulation. Clarifying how ID affects income distribution across different regions and development stages will offer valuable theoretical support for further promoting urban-rural integrated development and achieving inclusive prosperity goals.

With the accelerated development of digital technologies, academia has engaged in extensive and diverse discussions on their impact on the urban-rural income gap. Some studies suggest that improvements in digital infrastructure, internet penetration, and digital financial coverage are enhancing rural economic vitality by elevating rural human capital and income levels, thereby narrowing the urban-rural income gap to some extent [10–14]. However, another series of studies points out that the early benefits of digital technologies usually prioritize urban residents, particularly those with higher technical literacy and resource accessibility-thus exacerbating the urban-rural income gap [15,16]. Intelligent and automated technologies significantly raise wage growth rates for highly skilled workers, who are more concentrated in urban areas, thereby expanding regional income inequality [17,18]. Furthermore, some scholars propose a “U-shaped” effect: digital technologies initially widen the urban-rural gap, but as their penetration deepens in rural areas, the gap may gradually narrow [19,20].

It is noteworthy that inconsistencies in existing research findings largely stem from differing interpretations and definitions of the broad concept of “digital technology.” As digital technology spans multiple dimensions, researchers have approached the topic from varying perspectives and adopted diverse measurement frameworks. Most current studies focus on digital infrastructure, financial inclusion, and individual-level digital skills, examining how these factors influence the evolution of the urban-rural income gap by enhancing rural economic capacity, improving access to financial services, and strengthening human capital accumulation. In contrast, systematic research from the perspective of “industrial digitalization” remains limited. As a key manifestation of the deep integration of technological progress into the economic system, ID provides a novel analytical framework for understanding urban-rural income disparities. Therefore, this paper seeks to fill this gap by systematically examining the impact of ID on the urban-rural income gap.

This paper systematically examines the impact of ID on the urban-rural income gap from the perspective of ID. Through mechanism analysis and empirical testing, this study identifies two key channels: the development of software and information technology services, and the income gap between agricultural migrants and urban migrant populations. It further reveals how ID might intensify urban-rural income differentiation by increasing the concentration of high-end service industries and widening income disparities among migrant populations. Meanwhile, this paper conducts heterogeneity analysis across eastern, central, and western regions, comparing policy environments and structural conditions in different regions during the digital industry development process, aiming to expand theoretical explanations and empirical evidence for urban-rural income distribution issues within the context of the digital economy.

The main contributions of this study are threefold. First, regarding theoretical mechanisms, this paper explores how ID affects the urban-rural income gap, particularly through two pathways: the development of the software and information technology services industry and income disparities among migrant populations. The research shows that ID, by promoting software and information technology services, increases employment in the information services sector and raises software business revenue. Urban residents benefit more due to their educational and skill advantages, while rural residents benefit less, further widening the urban-rural income gap. Additionally, ID exacerbates the income gap between agricultural migrants and urban migrants, further enlarging the urban-rural income disparity. Second, from the empirical analysis perspective, this paper uses panel data from 30 Chinese provinces between 2012 and 2022 and employs a fixed-effects model to empirically examine the relationship between ID and the urban-rural income gap. The results indicate that ID significantly widens the urban-rural income gap. Finally, in terms of heterogeneity analysis, this study compares impacts across different regions and finds that, in the eastern region, due to the advantages of digital infrastructure, the advancement of ID significantly widens the urban-rural income gap. In contrast, in the central and western regions, the impact of ID on the urban-rural income gap is insignificant because of relatively lower digital development levels.

The remainder of this paper is structured as follows: Section 2 reviews relevant literature and proposes research hypotheses; Section 3 introduces the research design, including variable definitions, data sources, and model specifications; Section 4 presents the empirical analysis verifying the impact of ID on the urban-rural income gap; Section 5 discusses the mechanisms involved, identifying the transmission paths through the information services industry and income disparities among migrant populations; and Section 6 summarizes research conclusions, proposes policy recommendations, and discusses research limitations.

## 2 Theoretical analysis and research hypotheses

The wave of ID has created unprecedented opportunities for modern society, fundamentally altering traditional industry operation modes and giving rise to many new economic forms and service models [21]. However, this process has not progressed uniformly across all regions; significant developmental imbalances between urban and rural areas have emerged due to existing divides [22]. This imbalance is particularly evident in infrastructure construction, technological resource accessibility, and human resource allocation, which together result in a notable widening of the urban-rural income gap.

Urban areas, as central hubs of economic activities and technological innovation, are well-developed with digital infrastructures such as Internet facilities, high-speed broadband networks, and data centers, providing strong support for urban manufacturing, service industries, and other sectors [23]. By incorporating cutting-edge technologies such as big data analytics, artificial intelligence, and the Internet of Things, urban enterprises can optimize their production processes, enhance product and service quality, and explore emerging markets like e-Commerce and sharing economy [24]. These advancements not only enhance the competitiveness of enterprises, but also create more high-skilled, high-paying jobs opportunities for urban residents, thereby raising overall income levels.

In contrast, rural areas, often geographically remote and economically less developed, frequently face inadequate digital infrastructure [25]. High-speed Internet access is not yet fully available in many rural areas, and the application of information and communication technologies remains relatively limited, restricting opportunities for agricultural and other traditional industries to transition toward intelligence and automation. Due to insufficient technological support, rural industries struggle to align precisely with market demands, limiting improvements in the added value of their products. Moreover, rural education and training systems have lagged digital trends, resulting in local labor forces lacking essential new skills and impeding their participation in higher-level economic activities.

With advancements in digital technologies, especially the application of emerging technologies such as the Internet, big data, and artificial intelligence, significant transformations have occurred in agricultural production and non-agricultural employment sectors [26]. For individuals who can effectively utilize these technologies, unprecedented opportunities have arisen to enhance productivity, optimize resource allocation, and access new markets. However, the benefits of these technologies are not evenly distributed. Urban residents typically have better access to educational resources and technical training, enabling them to better adapt to new technologies and occupy the high-value-added jobs resulting from digital transformation. Conversely, rural areas, with lower educational attainment and technological adoption levels, see residents predominantly engaged in low-skilled, low-paid employment when participating in non-agricultural jobs. Even when rural residents attempt to improve household income through non-agriculture employment, they face higher search costs and risks compared to their urban counterparts. More importantly, the existence of a “secondary digital divide”, referring to disparities between urban and rural residents in their ability to apply digital technologies, not only prevents rural residents from fully benefiting from ID, but also potentially leads to a “Matthew effect”, where the strong get stronger, the weak get weaker. Over time, this gap may self-reinforce, continuously widening wage disparities between high-skilled and low-skilled workers, ultimately exacerbating the urban-rural income gap. Therefore, the following hypothesis is proposed:

H1: The development of industrial digitalization in China expands the urban-rural income gap.

The software and information technology services industry refers to activities involving processing and providing information services through technologies such as computers. It mainly includes software development, information systems integration services, information technology consulting services, data processing and storage, integrated circuit design, and other IT services [27].

With the accelerated advancement of ID, enterprises in traditional industries increasingly rely on digital technologies to pursue higher efficiency and competitiveness, significantly influencing the software and information technology services industry. On one hand, it has created substantial new demands, such as intelligent manufacturing and supply chain optimization solutions for manufacturing industries; on the other hand, it has driven technological innovation and the transformation of service models. Service providers must continuously launch more intelligent and customized products, gradually shifting from traditional software sales to cloud-based services and subscription models, emphasizing user experience and service quality [28].

The development of the software and information technology services industry exacerbates urban-rural income disparities [29]. As more enterprises adopt digital solutions, the industry not only expands its scope of services, but also significantly improves quality through high-end technical services like cloud computing, big data analytics, and artificial intelligence, thereby achieving remarkable income growth. As a knowledge-intensive industry, the software and information technology services sector set high entry barriers, requiring practitioners to possess substantial educational backgrounds and technical skills, including but not limited to professional IT training, higher education attainment, and familiarity with the latest digital tools and platforms. Urban areas, typically offering superior educational resources, more vocational training opportunities, and more developed digital infrastructure, enable urban residents to more readily acquire qualifications and skills necessary for the IT services sector. Consequently, the growth of this industry disproportionately benefits urban populations who are already economically advantaged, enhancing their salaries and employment prospects.

Additionally, the industry’s development is accompanied by automation and intelligent technologies, further reducing simple repetitive tasks. Traditionally, migrant workers, concentrated in labor-intensive sectors like manufacturing, construction, and low-end services, perform many repetitive jobs. However, advances in software algorithms, robotic process automation (RPA), and other intelligent technologies increasingly replace such jobs [30]. Migrant workers lacking essential retraining and reeducation opportunities lose critical income sources and struggle to transition quickly to high-value-added service positions. Conversely, well-educated urban workers adapt more rapidly to emerging job demands and benefit from the growth in the information services industry. This asymmetric employment restructuring directly exacerbates income inequalities between urban and rural areas. Therefore, the following hypothesis is proposed:

H2: Industrial digitalization expands the urban-rural income gap by promoting the development of software and information technology services industry, increasing software business income and employment in IT services.

Within urban-rural factors flow, changes in migrant population income structures constitute a critical mechanism for understanding income disparities. Particularly amid deepening ID, the economic status and income levels of migrant populations are significantly affected. Migrant populations primarily include agricultural migrant workers and urban migrants, with fundamental differences in human capital characteristics, employment structures, and social integration [31].

Agricultural migrants refer to individuals holding rural household registration who migrate to cities for employment. They generally have lower educational attainment, inadequate skills training, and limited social resources, typically engaging in low-value-added, labor-intensive sectors within urban labor markets. Facing digital transformation, agricultural migrants often struggle to adapt to new skill requirements imposed by the digital economy, limiting their income growth potential. As urban digital industries expand rapidly, income levels of highly skilled, well-educated groups rise sharply, marginalizing agricultural migrants due to their skill disadvantages. This income disparity reduces their opportunities for economic improvement, exacerbating urban-rural income gaps.

Urban migrants, who typically hold urban household registration and migrate within or across cities, usually possess higher educational levels and stronger skills, adapting more readily to digital economic demands. However, given continuously rising skill requirements in digital sectors, income growth among urban migrants still faces challenges. Although less severely impacted than agricultural migrants, they have not yet substantially benefited from digitalization in the short term [32]. Thus, the income enhancement effect of ID on urban migrants may be relatively limited. ID suppresses income growth for agricultural migrants without significantly increasing urban migrants’ income levels, further exacerbating urban-rural income disparities. Based on the above theoretical analysis, the following hypothesis is proposed:

H3: Industrial digitalization expands the urban-rural income gap by reducing the income levels of agricultural migrants without significantly affecting urban migrant incomes.

Based on geographical location and economic development levels, this study divides the 30 provinces of China into three regions: eastern, central, and western.

In the economically advanced eastern region, the urban-rural income gap tends to widen as the level of ID increases. This region boasts relatively well-developed digital infrastructure and technological resources, along with close connections between urban and rural areas. As ID advances, urban areas are more capable of adopting and leveraging new technologies quickly, thereby creating more high value-added employment opportunities and accelerating urban economic growth [33]. In contrast, rural areas often lag in digital adoption, which exacerbates income disparities between urban and rural residents. Therefore, in the eastern region, where development is relatively high, ID significantly widens the urban-rural income gap.

In the central region, although recent years have seen improvements in digital infrastructure driven by policy support and investment, the industrial structure still largely relies on traditional manufacturing and basic service industries. The penetration of digital technologies into core industries remains limited, constraining the role of ID in promoting economic growth and employment restructuring. Additionally, rural areas in the central region still lag in digital skills and information technology application capabilities. As a result, differences in the ability of urban and rural residents to benefit from the digital economy have not significantly widened. The limited employment and income gains driven by digitalization are mainly concentrated in a few urban core areas. Consequently, ID has not yet had a significant effect on the urban-rural income gap in the central region, which is reflected in the regression results as a statistically insignificant relationship.

In the western region, ID is constrained by complex geographical conditions and a weak economic foundation. The level of digital infrastructure construction is generally low, and the pace of digital transformation lags markedly [34]. At present, ID in the western region is mostly concentrated in a few medium and large cities, while rural areas still face significant shortcomings in infrastructure, educational resources, and technology application. As a result, widespread adoption and deep integration of ID in rural areas remain difficult. Moreover, because both urban and rural areas in the west generally start from a low base in terms of digital development, the limited digital progress to date has not been sufficient to significantly alter the overall urban-rural income structure. Therefore, at the current stage, the impact of ID on the urban-rural income gap in the western region is not yet apparent, and the regression results reflect this insignificance. Based on the above theoretical analysis, the following hypothesis is proposed:

H4: In China’s eastern region, ID significantly widens the urban-rural income gap, whereas in the central and western regions, its impact on the urban-rural income gap is not significant.

## 3 Methods and data

This chapter mainly introduces the research methods and data used in this study, focusing on three aspects: model specification, variable definition, and data sources. It aims to clarify the technical approach and data foundation upon which the empirical analysis is based, thereby laying the methodological groundwork for the subsequent regression analysis and mechanism testing.

### 3.1 Modeling specification

To determine the appropriate model specification, this study conducted a Hausman test. The test result (P = 0.0563) rejected the null hypothesis of the random effects model. Therefore, the fixed effects model was selected as the baseline regression method to investigate the impact of ID on the urban-rural income gap. This model controls both time-invariant regional characteristics and region-invariant temporal characteristics, effectively reducing omitted variable bias and improving the robustness of the estimation results. The specific model is set as follows:


$$Theil_{it}=\alpha_{0}+\alpha_{1}Idig_{it}+\beta Control+\mu_{i}+\nu_{t}+\varepsilon_{it}$$ (1)

Where Theil denotes the urban-rural income gap; Idig is the level of ID; Control is a vector of control variables, including the degree of openness to the outside world (Open), the intensity of fiscal support (Fina), R&D intensity (R&D), level of social consumption (Cons), level of economic development (lnGDP), level of education (Edu), and population mobility trends (Mig). i and t represent the individual provinces and the year, respectively; $\alpha_{1}$ is the coefficient of the level of ID; and β is a vector of coefficients for the series of control variables. $\mu_{i}$ and $\nu_{t}$ are the coefficient of ID level. $\varepsilon_{it}$ is the random error term that follows a normal distribution.

### 3.2 Selection of variables

#### 3.2.1 Dependent variable: Urban-rural income gap.

Common indicators for measuring the urban-rural income gap include the Gini coefficient, Theil index, and the ratio of per capita disposable income between urban and rural residents. The Gini coefficient is an internationally recognized tool for measuring overall income inequality and can effectively reflect the distribution of social income. However, it mainly focuses on general income distribution and lacks the capacity to distinguish structural income disparities between urban and rural areas. The ratio of per capita disposable income between urban and rural residents is simple to compute and intuitively reflects the direct income gap between the two groups, but it fails to fully account for differences in population structure.

In contrast, the Theil index not only measures overall income disparity but also decomposes the total disparity into within-group and between-group differences. It effectively considers the dual heterogeneity of income levels and population size, offering strong decomposability and dynamic analytical capabilities. As such, it can more accurately capture the changing characteristics of the urban-rural income gap [35]. Therefore, this study adopts the Theil index as the primary indicator for measuring the urban-rural income gap, aiming to enhance the scientific rigor and explanatory power of the analysis. The formula for calculating the Theil index is:


$$Theil\;\;\;Index_{it}=\sum_{i=1}^{2}\left(\frac{I_{it}}{I_{i}}\right)\ln\frac{I_{it}/Pit}{I_{i}/P_{i}}$$ (2)

In this study, $I_{1t}$ denotes the income of urban residents in year t, and $I_{2t}$ represents the income of rural residents in year t. The variable $I_{i}$ refers to the ratio of total income between urban and rural residents, while $P_{t}$ represents the ratio of their total population. A higher Theil index indicates a greater disparity in income between urban and rural areas. For robustness testing, the urban-rural income ratio is employed as an alternative explanatory variable.

#### 3.2.2 Core explanatory variable: Industrial digitalization.

In June 2020, the Department of Informatization and Industrial Development of the State Information Center of China officially defined the concept of “industrial digitalization” for the first time, proposing a framework based on information networks as the foundation, data resources as the key element, and scenario applications as the core. Despite growing academic interest, there is still no universally accepted standard for measuring ID. Some researchers adopt single indicators such as the digital penetration rates of the three major industries published by the China Academy of Information and Communications Technology, or the number of industrial robots, to proxy digitalization. However, due to the multi-faceted nature of ID, relying on single-dimensional indicators often leads to measurement bias.

Composite indicator systems, which can capture ID from multiple angles, have become a mainstream method in recent years. At the industrial level, studies commonly start from secondary indicators such as direct consumption coefficients and complete dependency ratios to estimate digital input intensity. At the regional level, particularly in provincial studies, researchers generally adopt multi-dimensional evaluation frameworks, considering factors such as digital talent, digital investment, digital output, and digital infrastructure. Drawing upon prior research and considering data availability and measurability, this paper constructs a comprehensive evaluation system based on four primary dimensions: digital infrastructure, digital talent, digital investment, and digital output, with a total of 12 secondary indicators used for measurement [36] (see Table 1).

**Table 1:** ID evaluation indicator system.

Primary Indicator	Secondary Indicator	Attribute
Digital Infrastructure	Long-distance optical cable length per unit area (km/km²)	Positive
	Broadband internet users per resident (10,000 households/10,000 people)	Positive
	Mobile phone penetration rate (%)	Positive
	Broadband access ports per capita (10,000 ports)	Positive
	Mobile phone users per 100 people (units/100 people)	Positive
Digital Talent	Internet users per 100 people (households)	Positive
	Employment in information transmission, computer services, and software sector per employed population (10,000/10,000)	Positive
Digital Investment	R&D staff in large-scale industries per resident population (10,000/10,000)	Positive
	Digital Inclusive Finance Index	Positive
Digital Output	Patent applications per employed population (items/10,000 people)	Positive
	Number of AI enterprises (units)	Positive
	Telecom service revenue per capita (CNY/person)	Positive

Among these dimensions, digital infrastructure reflects the regional allocation of digital resources and the extent of network coverage, serving as the foundational condition for digital transformation. Digital talent captures the stock and quality of human capital that supports the digital transformation of industries, focusing on the availability of information technology professionals and related technical personnel. Digital investment represents the financial and labor resources that a region allocates to research and development as well as innovation, reflecting the intensity and sustainability of its digital transformation efforts. Finally, digital output refers to the economic returns generated through digital economic activities, highlighting the productivity improvements and value creation brought about by digitalization processes. To ensure the comparability and standardization of the selected indicators, all variables were normalized. The entropy method was then applied to assign objective weights to each indicator, thereby minimizing bias introduced by subjective weighting. This approach enables a more scientific and comprehensive assessment of the level of ID across provinces.

#### 3.2.3 Control variables.

This study selects several variables that significantly affect the urban-rural income gap as control variables, including the degree of opening up to the outside world (Open), the strength of financial support (Fina), the intensity of R&D (R&D), the level of social consumption (Cons), and the level of economic development (lnGDP), education level (Edu), and population mobility trends (Mig).

The degree of openness is closely related to the urban-rural income gap. Controlling for openness helps eliminate the interference of differences in international trade activities, allowing for a more accurate estimation of the independent impact of ID on the income gap between urban and rural areas [37]. Fiscal support intensity reflects the government’s role in improving public services and supporting programs for low-income groups. It helps smooth income fluctuations and improve living conditions, thereby directly contributing to the narrowing of the urban-rural income gap [38]. R&D intensity, measured by the ratio of R&D expenditure to GDP, is an important indicator of a region’s innovation capacity. By controlling R&D intensity, we can eliminate the impact of differences in technological progress and innovation activities, allowing for a clearer assessment of the independent effects of ID. Level of social consumption indicates the total value of goods and services consumed by residents, and serves as an important indicator of both economic development and social welfare. It reflects changes in consumption capacity and living standards, which indirectly influence the income gap. Level of economic development, typically measured by per capita GDP, directly affects the quality of income and employment opportunities. Controlling for this variable helps eliminate distortions caused by different stages of regional development. Education level influences individuals’ employment capabilities and income potential. Regions with higher educational attainment are better positioned to meet the skill requirements of ID. Consequently, accounting for education attainment isolates the impact of human capital differences on income disparity. Population mobility trends directly affect the efficiency of talent allocation under digitalization. These trends determine the speed and depth of digital technology penetration across regions. Meanwhile, spatial redistribution of the population reshapes the urban-rural labor structure and significantly affects income distribution patterns. Thus, population mobility is an essential factor that must be controlled when analyzing the urban-rural income gap.

By controlling these variables, it is possible to exclude other factors from interfering with the results, making the regression analysis more robust and ensuring that the conclusions obtained primarily reflect the effect of ID on the urban-rural income gap, rather than the results of other factors. All variable definitions and descriptions are shown in Table 2.

**Table 2:** Variable definition and description.

Type	Definition	Notation	Variable Description
Dependent Variable	Urban-Rural Income Gap	Theil	Ranges from 0 to 1; higher values indicate greater income inequality.
Core Explanatory Variable	Industrial Digitalization (ID)	Idig	Higher values indicate a higher level of ID.
Mechanism Variable	Employment in Information Services	lnNumSer	Log-transformed
	Software Business Revenue	SofRev	Software business revenue as a share of regional GDP
	Income of Agricultural Migrants	InRu	Log-transformed
	Income of Urban Mobile Population	InUr	Log-transformed
Control Variable	Degree of Openness	Open	Total imports and exports as a share of regional GDP
	Fiscal Support Intensity	Fina	General budget expenditure as a share of regional GDP
	R&D Intensity	R&D	Internal R&D expenditure as a share of regional GDP
	Social Consumption Level	Cons	Total retail sales of consumer goods as a share of regional GDP
	Level of Economic Development	lnGDP	Log of per capita regional GDP
	Education Level	Edu	Number of students per resident population
	Population Mobility Level	Mig	Resident population to registered population ratio

### 3.3 Data sources

This study conducts an empirical analysis using panel data from 30 Chinese provinces for the period 2012–2022. Due to severe data unavailability, Hong Kong, Macao, Taiwan, and Tibet are excluded from the sample, in line with prior research in the field of urban-rural income disparity [39,40].

Data for dependent variable, urban-rural income gap, including per capita disposable income of urban and rural residents and the total population of urban and rural residents, are sourced from the China Statistical Yearbook. The core explanatory variable, ID, draws data from the Digital Finance Research Center of Peking University, China Industrial Statistical Yearbook, China Statistical Yearbook, and individual Provincial Statistical Yearbooks. The variables employed in regression analysis constitute a structured panel dataset (main_data.dta), covering annual observations from 2012 to 2022.

Data for mechanism variables such as the number of employees in the information services sector, software business revenue, income of agricultural migrant workers, and income of urban mobile population are collected from the China Statistical Yearbook, CSMAR database by GTA, and monitoring data on floating population from the National Health and Family Planning Commission. Individual-level income data for migrant populations constitute the micro- dataset incomeCMDS.dta, serving as the empirical basis for group-level heterogeneity analysis.

Data for control variables—degree of openness, fiscal support intensity, R&D intensity, level of social consumption, level of economic development, education level, and population mobility trends are also primarily obtained from the China Statistical Yearbook.

This study employs panel data from 30 Chinese provinces over the period 2012–2022 for empirical analysis, as detailed in S1 ProvinceData. The descriptive statistics of the main variables are presented in Table 3. It can be observed that the Theil index, which measures the urban-rural income gap, exhibits a substantial range between its minimum and maximum values. This indicates significant disparities in urban-rural income gaps across provinces during the 2012–2022 period. Specifically, the maximum Theil index corresponds to Guizhou Province in 2012, reflecting the highest urban-rural income disparity, while the minimum value is observed in Tianjin in 2022, indicating the smallest disparity. Similarly, the range of values for the ID index is also wide, suggesting considerable variation in the level of ID development among provinces during the same period.

**Table 3:** Descriptive statistics of the main variables.

Variable	Sample Size	Mean	Standard Deviation	Min	Max
Theil	330	0.083	0.037	0.017	0.197
Idig	330	0.179	0.117	0.024	0.593
lnNumSer	330	2.696	1.155	−0.693	5.308
SofRev	330	0.049	0.070	0	0.541
InRu	518469	−0.1979	0.1987	−1.1347	0.6968
InUr	107131	−0.1249	0.2825	−1.1147	1.6968
Open	330	0.265	0.268	0.008	1.354
Fina	330	3.465	1.082	1.805	7.618
R&D	330	0.017	0.011	0.002	0.065
Cons	330	0.393	0.066	0.180	0.610
lnGDP	330	10.908	0.445	9.849	12.155
Edu	330	0.0006	0.0007	0.0001	0.0031
Mig	330	1.0402	0.1986	0.8307	1.7147

## 4 Empirical results analysis

### 4.1 Baseline regression results

To test the hypothesis, the sample data are applied into Equation (1) to calculate the test statistic. Columns (1) and (2) of Table 4 represent the effects of ID on the urban-rural income gap before and after the inclusion of control variables, respectively. The baseline regression results indicate that, prior to the inclusion of control variables, a one-unit increase in the level of ID leads to a 0.106-unit increase in the urban-rural income gap, and this effect is statistically significant at the 1% level. This suggests that, when other factors are not considered, the development of ID significantly exacerbates the urban-rural income gap. After the inclusion of control variables, the magnitude of this effect decreases to 0.087 units per unit increase in ID, but the coefficient remains statistically significant at the 1% level. Although the coefficient decreases, the positive relationship remains robust, indicating that even after accounting for other factors that may affect income inequality, the rise in ID continues to widen the urban-rural income gap. The estimation results based on the fixed effects model show that the development of ID in China significantly increases the urban-rural income gap, thus confirming Hypothesis 1. Moreover, the inclusion of control variables enhances the robustness of this relationship, further validating the reliability of the conclusion.

**Table 4:** Baseline regression results.

	(1)	(2)
	Theil	Theil
Idig	0.106***	0.087***
	(3.939)	(4.086)
Open		-0.021***
		(−2.898)
Fina		0.006*
		(2.019)
R&D		-0.073
		(−0.728)
Cons		-0.013
		(−1.262)
lnGDP		-0.024
		(−1.697)
Edu		18.186*
		(1.994)
Mig		-0.025
		(−1.314)
Individual and Time Fixed Effects	Controlled	Controlled
Constant	0.095***	0.364**
	(38.546)	(2.331)
N	330	330
R²	0.869	0.922

Note: ***, **, and * indicate significance at the 1%, 5%, and 10% levels, respectively. t-values are in parentheses. Same applies to the following tables.

In the model where industrial digitization significantly exacerbates urban-rural income gap, control variables such as R&D intensity, social consumption level, economic development level, and population mobility level exhibit statistical insignificance. This phenomenon primarily stems from industrial digitization’s dominant role in directly intensifying income polarization through the “digital divide,” whose influence substantially overshadows other variables. Specifically, rural R&D investments struggle to translate into productivity due to infrastructure deficiencies and talent shortages. Digital consumption displays a polarized trend, where urban residents disproportionately benefit from digital service consumption while rural consumption remains necessity-driven, thereby weakening the equalizing potential of consumption upgrades. Highly developed regions may have crossed a digital “turning point” where income distribution effects plateau, whereas less developed areas lack scale effects. Linear models fail to capture this nonlinear complexity in economic development impacts. Short-term, low-skilled labor mobility faces digital skill mismatches, hindering entry into high-value-added positions and counteracting mobility’s inherent equalizing potential.

### 4.2 Endogeneity test

This study may face potential endogeneity issues, such as the omission of variables that significantly influence regional differences, or the existence of a bidirectional causal relationship between the level of ID and the urban-rural income gap. To address these concerns, the study employs instrumental variable (IV) techniques by using the one-period lag of the explanatory variable, as well as an interaction term between the 1984 number of fixed telephone lines in each province and the lagged ID level, as instruments for mitigating endogeneity.

#### 4.2.1 Test using lagged explanatory variable.

Since the prior level of ID is unlikely to be affected by the current urban-rural income gap, the one-period lag of ID is used as an instrumental variable in a two-stage least squares (2SLS) regression to alleviate potential reverse causality. The estimation results are presented in Table 5.

**Table 5:** Two-Stage Least Squares Results Using Lagged ID as Instrumental Variable.

Variable	First Stage	Second Stage
	Level of ID (Idig)	Rural-urban income disparities (Theil)
Idig		0.090***
		(3.091)
L.Idig	1.061***	
	(34.774)	
Open	0.018*	−0.055***
	(1.874)	(−4.463)
Fina	-0.003	−0.001
	(−1.634)	(−0.006)
R&D	0.149	0.137
	(0.937)	(0.638)
Cons	0.018	−0.012
	(1.262)	(−0.478)
lnGDP	-0.003	−0.063***
	(−0.497)	(−7.036)
Edu	1.647	1.891
	(1.000)	(0.917)
Mig	-0.007	0.011
	(−0.538)	(0.612)
Individual and Time Fixed Effects	Controlled	Controlled
K-P rk LM	43.400***	
K-P F	1209.242	
Adj.R2		0.616

In the first-stage regression, the lagged ID level (L.Idig) shows a significantly positive effect on the current level of ID. The K-P rk LM and K-P F are both significant at the 1% level, confirming the relevance of the instrumental variable. In the second-stage regression, the impact of ID on the urban-rural income gap remains significantly positive after using the instrument, suggesting that ID continues to widen the income gap. This indicates that even after addressing endogeneity concerns, the conclusion of this study remains robust and credible.

#### 4.2.2 Instrumental variable test.

Selecting appropriate instrumental variables to replace the core explanatory variable is an effective method for addressing endogeneity. Drawing on existing literature, this study uses the number of fixed telephone lines in each province in 1984 as an instrument to evaluate digital transformation. Since traditional communication technologies serve as the foundation for digital development, provinces with higher historical fixed-line penetration tend to have more advanced telecommunications infrastructure, which facilitates the development of ID. This satisfies the relevance condition for a valid instrumental variable.

Given that the fixed-line data are cross-sectional, this study follows the approach of Liu et al. [41], constructing an interaction term between the number of fixed telephone lines in 1984 and the lagged level of ID as the instrumental variable for digital transformation. Table 6 reports the estimation results from the two-stage least squares (IV-2SLS) method. The regression coefficients for the explanatory variable remain significantly positive, indicating that the effect of ID in widening the urban-rural income gap continues to hold. The Kleibergen-Paap rk LM statistic reports a p-value of 0.000 for both instruments, strongly rejecting the null hypothesis of under-identification. Furthermore, the Kleibergen-Paap rk Wald F statistics exceed the critical value at the 10% level of the Stock-Yogo weak identification test, indicating that weak instruments are not a concern. These tests collectively demonstrate the scientific validity and appropriateness of the selected instruments, and the regression results further confirm that the core findings of this study remain robust and reliable.

**Table 6:** Two-Stage Least Squares Results Using 1984 Fixed-Line Telephones as Instrumental Variable.

Variable	(1)	(2)
	Level of ID(Idig)	Rural-urban income disparities(Theil)
Idig		0.086**
		(2.001)
L.Idig	0.012***	
	(4.015)	
Open	0.052***	−0.055***
	(2.713)	(−4.278)
Fina	0.014***	0.001
	(3.789)	(0.012)
R&D	-0.330	0.137
	(−0.902)	(0.638)
Cons	0.076**	−0.012
	(2.332)	(−0.467)
lnGDP	0.120***	−0.063***
	(8.042)	(−6.784)
Edu	1.747	1.867
	(0.468)	(0.909)
Mig	0.029	0.012
	(0.921)	(0.616)
Constant	−1.304***	0.761***
	(−8.840)	(8.048)
Individual and Time Fixed Effects	Controlled	Controlled
K-P rk LM	25.425***	
K-P F	16.121	
Observations	300	300
Adjusted R²		0.616

### 4.3 Robustness tests

To verify the robustness of the baseline regression results, this study employs two alternative methods: (1) replacing the dependent variable, and (2) re-estimating the model using the System GMM approach.

#### 4.3.1 Replacing the dependent variable.

The dependent variable is replaced by the urban-to-rural per capita income ratio instead of the urban-rural income gap measured by the Theil index. The regression results are reported in Column (1) of Table 7. The results show that the level of ID still has a significantly positive effect on the urban-to-rural income ratio, indicating that the development of ID continues to widen the income gap between urban and rural residents. This finding confirms the robustness of the baseline regression results.

**Table 7:** Robustness tests.

	(1)	(2)
	Substitution of explanatory variables	GMM model
L.Gap		0.940***
		(35.808)
Idig	0.712***	0.018*
	(4.218)	(1.877)
Open	-0.078	0.007***
	(−1.122)	(3.032)
Fina	0.055**	−0.001
	(2.263)	(−0.574)
R&D	0.451	0.041
	(0.478)	(1.114)
Cons	-0.006	−0.001
	(−0.053)	(−0.167)
lnGDP	-0.170	−0.007
	(−1.274)	(−1.065)
Edu	145.369	−0.495
	(1.120)	(−0.437)
Mig	-0.229	−0.004
	(−0.870)	(−0.370)
Individual and time Fixed Effects	Controlled	Controlled
Constant	4.454***	0.070
	(2.961)	(1.126)
N	330	300
R²	0.895	

#### 4.3.2 Using the system GMM model.

To further address potential endogeneity and account for the dynamic adjustment characteristics of the urban-rural income gap, this study adopts the System Generalized Method of Moments (System GMM) for model re-estimation. System GMM effectively addresses the endogeneity of explanatory variables and improves estimation efficiency by using lagged variables as instruments [42]. The regression results show that the AR (1) test is significant while the AR (2) test is not, indicating that the model does not suffer from second-order autocorrelation. Furethermore, the Hansen test supports the validity of the instrumental variables. Therefore, the System GMM estimation further reinforces the core conclusion that ID exacerbates the urban-rural income gap. The results are presented in Column (2) of Table 7.

### 4.4 Heterogeneity test

The development of ID is closely tied to a region’s level of economic development, which inevitably affects the urban-rural income gap. Given this, the effects of ID may vary across different regions of China. To examine potential regional heterogeneity, this study divides the 30 provinces into three groups and conducts sub-sample regressions.

Specifically, this study divides China’s 30 provinces into three major regions for sub-sample analysis. The eastern region consists of 11 relatively developed provinces and municipalities: Beijing, Fujian, Guangdong, Hainan, Hebei, Jiangsu, Liaoning, Shandong, Shanghai, Tianjin, and Zhejiang. The central region includes 9 provinces—Chongqing, Anhui, Heilongjiang, Henan, Hubei, Hunan, Jiangxi, Jilin, and Shanxi, which are characterized by transitional economies and traditional industrial bases. The western region comprises 10 less-developed areas, including Gansu, Guangxi, Guizhou, Inner Mongolia, Ningxia, Qinghai, Shaanxi, Sichuan, Xinjiang, and Yunnan, where geographic and infrastructural constraints are more pronounced.

The regression results are presented in Table 8. In the eastern region, a higher level of ID significantly widens the urban-rural income gap. In the central and western regions, ID also shows a positive association with income inequality, but the coefficients are not statistically significant. Overall, the central region demonstrates some tendency toward an increasing income gap, though the effect remains unstable, while the effect in the western region is even weaker and economically marginal. A closer look reveals that the eastern region has a strong economic foundation, a mature industrial system, and well-developed digital infrastructure. Moreover, the local governments have actively promoted digital economy development through targeted policy guidance and resource allocation. These advantages have enabled rapid digital technology diffusion in urban areas, fostering the growth of emerging industries and increasing urban incomes. However, rural areas in the east still lag in education, digital skills, and access to technological resources. As a result, the benefits of digitalization are largely captured by urban residents, further widening the income gap between urban and rural areas.

**Table 8:** Heterogeneity regression results.

	(1)	(2)	(3)
	Eastern part	Central Region	Western region
Idig	0.052**	0.054	0.002
	(2.500)	(1.334)	(0.058)
Open	-0.017*	0.018*	−0.006
	(−2.103)	(2.002)	(−0.175)
Fina	0.002	0.018***	0.001
	(0.836)	(5.326)	(0.211)
R&D	-0.194***	−0.484	0.057
	(−4.070)	(−1.174)	(0.242)
Cons	-0.006	−0.017	−0.020
	(−0.302)	(−1.640)	(−1.518)
lnGDP	-0.000	0.005	−0.019
	(−0.025)	(0.646)	(−1.001)
Edu	25.154**	43.000	−8.143
	(2.861)	(1.244)	(−1.013)
Mig	-0.037	0.116	0.088**
	(−1.745)	(1.533)	(2.379)
Constant	0.108	−0.113	0.270
	(0.531)	(−0.906)	(1.212)
Individual and Time Fixed Effects	Controlled	Controlled	Controlled
N	121	99	110
R²	0.906	0.967	0.974

In the central region, the economy is at a moderate stage of development, dominated by traditional manufacturing and basic service industries. Although digital infrastructure has improved with national support, and digital economic development has emerged in some urban areas, the overall penetration and application of digital technology in rural areas remain limited. Consequently, disparities in access to digital resources and income-generating opportunities persist. While there is evidence that ID may widen the income gap, the effect is constrained by weaker economic foundations and an incomplete industrial transformation, and thus the result lacks statistical significance. The western region faces more severe constraints. Due to its challenging geography, relatively low economic development, and narrow industrial base, digital infrastructure in the west is generally underdeveloped. Furthermore, the gap between urban and rural areas in access to information, skills training, and technological resources is even more pronounced [43]. Despite increased policy support from both central and local governments to promote digital infrastructure and industrial transformation, limitations such as small market size and weak technological spillovers hinder the economic impact of ID [44]. As a result, the regression analysis shows that ID has only a marginal effect on the urban-rural income gap in the western region.

In conclusion, regional heterogeneity in the impact of ID on the urban-rural income gap stems from disparities in economic structure, policy implementation capacity, and digital infrastructure endowment. The eastern region, benefiting from robust economic foundations and strong policy support, has achieved rapid digital development, yet this process has intensified spatial inequality. In contrast, the central region exhibits transitional phase with unstable effects, while the western region, constrained by foundational weaknesses, has yet to translate ID investments into meaningful economic returns.

## 5 Mechanism test analysis

This section further explores the underlying mechanisms through which ID affects the urban-rural income gap, employing a mediation effect model to conduct mechanism testing. Following the theoretical framework presented earlier, we apply mediation analysis to examine how ID influences the urban-rural income gap. Drawing on suggestions from Alesina and Zhuravskaya regarding mediation analysis in causal inference, this study selects mediating variables with clear and intuitive causal links to the dependent variable (urban-rural income gap), and places particular emphasis on the effect of the explanatory variable on the mediators [45]. Based on the baseline regression model (1), the mediation test model (3) is constructed as follows:


$$Mediator_{it}=\alpha_{0}+\alpha_{1}Idig_{it}+\beta Control+\mu_{i}+v_{t}+\varepsilon_{it}$$ (3)

Where the definitions and measurements of the explanatory and control variables are consistent with the baseline regression, and Mediator refers to the mediating variable. The definition and measurement of each mediating variable will be introduced in the following subsections.

### 5.1 Employment in the information services sector

According to our theoretical analysis, the development of ID significantly increases employment opportunities in the information services sector. These opportunities primarily benefit urban residents who have access to better education and professional training, thereby enhancing their income and employment prospects. In contrast, rural residents often lack access to such resources and are thus less able to participate in this high-growth sector, which exacerbates the urban-rural income gap. In this study, the number of employees in the information services sector is measured by its logarithmic form.

The regression results in column (1) of Table 9, show that a significantly positive coefficient of the explanatory variable ID on employment in information services. This indicates that ID significantly boosts employment in the information services sector, thereby contributing to widening the urban-rural income gap.

**Table 9:** Mechanism Test results: Information Services Employment and Software Business Revenue.

	(1)	(2)
	lnNumSer	SofRev
Idig	7.736***	0.304***
	(3.609)	(2.846)
Open	-0.849	−0.086**
	(−0.794)	(−2.123)
Fina	-0.223	−0.011
	(−0.620)	(−1.094)
R&D	39.014**	−2.620***
	(2.323)	(−3.382)
Cons	-0.346	−0.002
	(−0.235)	(−0.056)
lnGDP	-1.270	0.003
	(−0.767)	(0.078)
Edu	-87.108	18.113
	(−0.119)	(0.599)
Mig	2.173	−0.219**
	(0.902)	(−2.207)
Constant	13.685	0.296
	(0.784)	(0.868)
Individual and Time Fixed Effects	Controlled	Controlled
N	330	330
R²	0.120	0.534

### 5.2 Software business revenue

As ID advances, the information services sector provides high-end technological services such as cloud computing, big data analytics, and artificial intelligence, which significantly increases overall industry revenue [46]. Urban residents, equipped with better education and training opportunities, are more likely to obtain well-paying jobs in this sector and benefit from the industry’s growth [47]. In contrast, rural areas lack such resources, making it difficult for residents to access high-value digital service jobs, thereby further widening the urban-rural income gap.

In this study, the level of software business revenue is measured by the ratio of software business revenue to regional GDP. A higher value of this variable indicates a more developed software and IT services sector. The regression results in column (2) of Table 9 show that the coefficient of Idig is significantly positive, suggesting that ID promotes the growth of the software and information technology services sector, which in turn contributes to a widening of the urban-rural income gap.

### 5.3 Income of agricultural migrants

In addition to the information services sector, ID may also influence the urban-rural income gap by altering the income structure of the mobile population. As the key medium for urban-rural factor mobility, changes in the income level of the mobile population not only reflect the degree of urban-rural economic integration but also significantly affect income distribution patterns. Specifically, the income mechanisms of agricultural migrants and urban mobile workers differ, making it necessary to examine their responses to digital transformation separately [48].

Due to limited education and skill levels, agricultural migrants are more vulnerable in the process of ID. As shown in column (1) of Table 10, an increase in ID significantly reduces the relative income of agricultural migrants. Specifically, for every unit increase in ID, the income gap between agricultural migrants and the local average wage widens by 792 units, and the effect is significant at the 1% level.

**Table 10:** Mechanism Test result: Income of Agricultural Migrants and Urban Mobile Population.

	(1)	(2)
	InRu	InUr
Idig	-0.088***	0.055
	(−4.783)	(0.664)
Open	0.121***	−0.080***
	(28.274)	(−4.742)
Fina	-0.009***	0.001
	(−4.782)	(0.107)
R&D	-0.330	−1.802**
	(−1.227)	(−1.976)
Cons	0.040***	0.114***
	(5.866)	(5.883)
lnGDP	-0.125***	−0.068***
	(−15.230)	(−2.837)
Edu	179.348***	140.201***
	(21.491)	(5.300)
Mig	0.032*	0.163**
	(1.653)	(2.498)
constant	1.028***	0.367
	(10.434)	(1.256)
Province and Time Fixed effects	Controlled	Controlled
N	518469	107131
R²	0.401	0.069

This suggests that although agricultural migrants have physically relocated to urban areas, they still face disadvantages in skills, education, and technological adaptability. As digital technologies rapidly penetrate urban industries and labor market skill requirements rise, these low-skilled, low-educated workers find it difficult to enter emerging digital sectors. Instead, they remain in low-value-added jobs or risk marginalization [49]. As a result, even after moving to cities, the income growth of agricultural migrants lags that of local residents, leading to a continued decline in their relative income level and further widening the urban-rural income gap.

### 5.4 Income of urban mobile population

Compared to agricultural migrants, the urban mobile population generally has higher education levels and stronger skill sets, making them better equipped to adapt to ID. Therefore, the relationship between their income and the urban-rural income gap may differ.

The regression results in column (2) of Table 10 indicate that the coefficient for the urban mobile population is not statistically significant, suggesting that, at this stage, ID has not had a noticeable impact on their relative income level. This may be due to two reasons: first, the urban mobile population already possesses a reasonable level of skills and education, giving them a better capacity to adapt to the digital labor market compared to agricultural migrants; second, although these workers can find stable employment in traditional sectors, they have yet to transition in large numbers to high-end digital jobs. Consequently, their income levels have not improved significantly in the short term. In addition, barriers to social integration and limited upward mobility further constrain their income growth potential [50].

In summary, the development of ID has significantly reduced the relative income of agricultural migrants, while its positive effect on the income of the urban mobile population has yet to materialize. Together, these dynamics contribute to a further widening of the urban-rural income gap and exacerbate income inequality within regions.

## 6 Conclusion and policy recommendations

### 6.1 Main findings

Based on panel data from 30 Chinese provinces between 2012 and 2022, this study investigates the impact of ID on the urban–rural income gap. The results reveal that ID significantly expands the income disparity between urban and rural areas. Specifically, the advancement of ID promotes the development of software and information technology services, increasing employment in the information services sector and boosting software-related revenues. However, due to their clear advantages in educational resources, technical skills, and employment opportunities, urban residents are better positioned to access the digital sector and reap its benefits. In contrast, rural residents, limited by skill gaps and resource constraints, struggle to share the dividends of digitalization, thereby exacerbating the income gap between urban and rural populations [51].

In addition, the income levels of the agricultural labor transfer population are negatively affected by ID, as their skills and educational backgrounds are often mismatched with the demands of digital industries. Meanwhile, no significant income changes were observed for urban floating populations, indicating that digitalization does not have a notable effect on improving their earnings [52]. Regionally, the eastern region experiences a significant widening of the urban–rural income gap due to its advanced digital infrastructure and capacity for rapid technological adoption. The impact in the central region is less pronounced, potentially due to unstable digital development and slow industrial restructuring. In the western region, the effect is negligible, mainly due to underdeveloped digital infrastructure. Overall, the influence of ID varies significantly across regions depending on infrastructure, technology adoption, and economic development levels, further intensifying regional disparities in income.

### 6.2 Policy recommendations

This study finds that ID tends to widen the urban–rural income gap and manifests strong heterogeneity across regions and population groups. This implies that one-size-fits-all policies are inadequate in addressing the structural divergence caused by uneven digital development. To mitigate the income inequality effects and promote urban–rural integration, policy interventions must be tailored across several dimensions, including resource allocation, educational supply, industrial planning, and labor structure.

(1) Improve institutional guidance and factor support mechanisms to prevent structural imbalance caused by digitalization

The baseline regression results show that ID exacerbates the urban–rural income gap primarily due to the uneven regional distribution of digital technology resources, supporting industries, and skilled labor [53]. To address this, it is recommended that central government enhance fiscal transfer mechanisms to support central, western, and rural regions, focusing on investments in broadband networks, computing infrastructure, and industrial internet facilities [54]. Simultaneously, national digital R&D platforms should be encouraged to expand into inland and non-core areas, fostering local enterprise transformation through technological spillovers and improving the integration of disadvantaged regions into digital industrial chains.

(2) Develop regionally differentiated digital literacy improvement paths based on economic fundamentals

Heterogeneity analysis reveals that the impact of digitalization on the urban–rural income gap is most significant in the eastern region, remains unstable in the central region, and is negligible in the western region, which is still in the early stages of digital development. Accordingly, human capital enhancement strategies should be region-specific and aligned with the economic and educational capacities of each area, focusing especially on rural capacity building [55]. Emphasis should be placed on digital literacy education for rural populations as a key entry point for bridging the digital dividend gap. For instance, the eastern region should extend high-quality educational resources to rural towns, strengthen IT courses and teacher training in primary and secondary schools, and improve the digital adaptability of rural youth. The central region should accelerate the establishment of regional vocational education centers and offer practical courses in digital manufacturing and platform operations to align with industry demands. The western region should prioritize the introduction of remote learning platforms and public training programs to address infrastructure and faculty shortages, thereby laying a foundation for digital capacity.

(3) Promote the orderly expansion of digital services to smaller cities and counties to enhance employment connectivity

Mechanism variable analysis shows that while the development of the software and IT services industry increases overall income levels, job opportunities are heavily concentrated in urban areas, limiting the participation of rural populations. To broaden the industry’s impact, it is recommended that government bodies implement regional guidance policies to encourage tech companies to establish customer support centers, data processing hubs, or remote delivery platforms in smaller cities and counties. Incentives such as guidance funds, office rent subsidies, and tax relief should be offered to enhance enterprise willingness to localize operations. Furthermore, a portion of public digital service procurement contracts should be allocated to non-core enterprises [56]. Supporting measures include establishing digital job service platforms and promoting remote jobs and online collaboration to increase rural residents’ participation and income opportunities [57].

Enhance the vocational transition capacity of the agricultural labor transfer population to alleviate structural mismatches caused by digitalization

Another mechanism analysis shows that the income levels of agricultural labor migrants have significantly declined, as their skillsets are often incompatible with digital industry requirements. To address this structural imbalance, it is recommended that agricultural migrants be prioritized in digital vocational training programs. Local technical schools and township adult education centers should offer short-cycle training courses focused on digital manufacturing, e-commerce operations, and platform services [58]. Enterprises should also be involved in course design and practical training, offering certifications, job recommendations, and employment matching services to trainees. For returning migrant workers and laborers residing in urban–rural fringe areas, targeted entrepreneurship guidance and policy support should be provided to facilitate their integration into digital economy sectors.

### 6.3 Limitations and future research directions

This study faces certain limitations in measuring the level of ID. Due to the absence of a unified and authoritative standard, the selection of measurement indicators in academic literature varies. This study builds its framework based on existing research, focusing on digital infrastructure, digital talent, digital input, and output. While this approach reflects the basic landscape of ID, it may not fully capture its complexity or regional variation. Additionally, data availability and consistency in statistical definitions may affect the precision of the results and should be further addressed in future research. Finally, due to the huge differences in industry types, talent structures, and digital technology levels in different provinces in China, the impact of ID on urban-rural income disparities needs to be carefully considered in different regions.

Future research could expand in the following directions: First, with the rapid evolution of emerging technologies such as AI and big data, the definition and mechanisms of ID are also changing. It is recommended to further explore the differentiated impacts of new technologies across regions and population groups. Second, given the varying levels and trajectories of digitalization across different industries, future studies could examine how sectoral heterogeneity mediates the effects of digitalization on the urban–rural income gap. Finally, efforts could be made to construct more detailed and dynamic indicators of digitalization to improve the accuracy of assessments and the precision of policy recommendations.

## Supporting information

S1 ProvinceData Provincial-level panel data for 30 Chinese provinces from 2012 to 2022 are utilized for empirical analysis in this study. (XLSX)

S2 Files Replication package in Stata format, include: 1.main_data.dta: Province panel dataset (2012–2022) containing core variables, mechanism variables, control variables. 2.incomeCMDS.dta: Micro-level dataset from the China Migrants Dynamic Survey (CMDS). 3. dofile.do: Stata 17.0 script for data loading, variable construction, and regression analysis.
(ZIP)
